# Assessing user perspectives on clinical pharmacogenomics consultation documentation: a user-centered evaluation

**DOI:** 10.3389/fphar.2024.1377132

**Published:** 2024-05-09

**Authors:** Nina Desai, Namratha Ravindra, Bradley Hall, Hana Al Alshaykh, Lauren Lemke, Eda Eken, Emily J. Cicali, Kristin Wiisanen, Larisa H. Cavallari, Khoa A. Nguyen

**Affiliations:** ^1^ Department of Pharmacotherapy of Translational Research and Center for Pharmacogenomics and Precision Medicine, College of Pharmacy, University of Florida, Gainesville, FL, United States; ^2^ Alfaisal University, Riyadh, Saudi Arabia; ^3^ Lifespan Health, Providence, RI, United States

**Keywords:** pharmacogenomics, consultation, user-centered evaluation, SOAP note, phenoconversion

## Abstract

The University of Florida Health Precision Medicine Program plays a crucial role in delivering pharmacogenomics (PGx) result notes to providers who request PGx testing. Despite this, there is currently a lack of a formal assessment of provider needs and established best practice design principles to guide the ongoing development of PGx result notes. This study aims to enhance the content and format of the PGx consult note at UF Health by incorporating valuable feedback from healthcare providers. Through in-depth user sessions involving 11 participants, we evaluated the usability of our consult note template. While overall satisfaction with the content was noted, specific sections, including those addressing phenoconversion and the medication list, were identified for revision to enhance clarity based on insightful provider feedback.

## 1 Introduction

Established in 2011, the Precision Medicine Program (PMP) at the University of Florida Health (UF Health) initially focused on integrating pharmacogenomics (PGx) into routine clinical practice (Johnson et al., 2013). The program adopted a comprehensive system-wide approach, including the generation of written consult notes by PGx pharmacists (pharmacist with specialized training in PGx) for providers ordering PGx tests or requiring assistance with result interpretation. These consult notes, disseminated through the EPIC^®^ electronic medical record (EMR) as clinical progress notes, have been instrumental in facilitating communication and collaboration.

The PMP PGx pharmacists utilize electronic means to inform ordering providers about the availability of consultation documents. Since its inception, the PMP clinical service has delivered 1970 consultation notes to 160 providers, catering to both clinical and research needs. Despite being designed for prescribers, no formal usability assessment has been conducted on these consult notes to enhance their effectiveness. Furthermore, guidance and literature reviews on PGx consult notes are limited, with the INGENIOUS trial (Eadon et al., 2016) (2016) being one of the few studies addressing concerns related to PGx consult notes. Notably, the trial highlighted issues such as information overload and the potential for overwhelming providers with PGx information. Recognizing the necessity of a formal assessment, it is essential to leverage provider feedback to gauge satisfaction and the efficacy of documentation. Without such evaluation, the success of pharmacogenomic implementation may be impeded. Usability research, focusing on the user experience (UX) by deeply understanding users’ needs, values, abilities, and limitations, emerges as a valuable tool to analyze provider feedback (Rosala; Yen and Bakken, 2011; Elchynski et al., 2021). This research can contribute to the enhancement of the entire PGx consultation process by informing the optimal content and format of PGx consult notes and fostering a better understanding of PGx. In pursuit of optimizing the usability of consult notes, our objective is to capture the perspective of provider needs, enhance the current content of PGx consult notes at UF Health, and guide future developments in PGx documentation.

## 2 Materials and methods

### 2.1 Study setting

The research was carried out at UF Health Shands Hospital, a substantial learning health system that utilizes EPIC^®^ as its electronic medical record (EMR). The implementation of PGx consult documentation within the hospital is led by the UF Health Precision Medicine Program (Johnson et al., 2013), a team primarily comprised of PGx specialist pharmacists. All procedures were developed in alignment with the clinical consult notes used at the initiation of the study. Approval for the study was granted by the University of Florida Health Quality Improvement Project Registry.

### 2.2 Participant recruitment

Each participant was invited via email to participate in our in-depth user sessions (1-3 participants per session). We recruited UF Health clinicians who had previously ordered PGx testing and been the recipient of a PGx results interpretation (using a report generated from PGx clinical decision support alerts). Each session was moderated by either a PGx pharmacy resident or a pharmacy student and lasted between 30 and 60 min. Each participant verbally consented prior to the session through a secure online video chat.

### 2.3 Moderator guide development

A standardized moderation guide (see supplemental document A) was collaboratively developed by a team comprising two PGx residents, a PGx pharmacist, an informatics pharmacist, and two pharmacy students. The guide was designed for use with PGx consult notes from UF Health. In our study, we sought feedback on the logic of the consult note to help improve the content and format of PGx consultation. Our PGx clinical service typically employs the SOAP format (Subjective, Objective, Assessment, and Plan), a widely adopted format across healthcare systems (Pearce et al., 2016). We called this format “traditional format”. In contrast, a flipped format, wherein the PGx test results and interpretation are positioned at the top of the note—an alternative format option preferred by the participants. At the time of study, both formats were implemented in clinical practice. Two sample notes (see supplemental document B) were incorporated into the moderation guide. Importantly, these notes were extracted from genuine patient PGx consult notes, ensuring compliance with HIPAA regulations by removing all patient identifiers. The flipped note illustrates a sample patient with a gastrointestinal case.

### 2.4 In-depth user sessions

Each sessions were led by 1-2 moderators and were recorded with the participants’ prior consent. Before each session, participants were sent a modified Computer System Usability Questionnaire (CSUQ) (Lewis, 2018), a validated computer usability satisfaction questionnaire via REDCap^®^ survey (UF Redcap, Nashville, TN). The CSUQ survey sought clinicians’ assessments of the current PGx consult note across various characteristics. We combined and presented data in seven categories: organization, ease of comprehension, information quality, clarity of the future medication and phenoconversion section, helpfulness, and overall satisfaction. We modified the questionnaire to replace “this system” by “this note” or “phenoconversion session” to improve the clarity of survey questions. Providers were presented with a set of statements and asked to express their opinions on each, ranging from strongly disagree to agree for each statement (Lewis, 2018). Additionally, the survey gathered information about participant demographics, their experience with EPIC^®^ EHR, and reflections on their knowledge of PGx.

During the sessions, participants were introduced to a set of two sample PGx consult notes, representing different clinical scenarios while adhering to the existing content and format. They were given a few minutes to familiarize themselves with each consult note. The moderator then initiated a series of predetermined and impromptu questions through a standardized script (see supplemental document A) to assess the participants’ ease of comprehension and application of pharmacogenomic information. The questions also delved into the appropriateness of specific sections’ presence and placement, such as relevant laboratory markers and the patient’s past medication list. This approach was employed to ensure consistency across sessions and maintain a structured exploration of participants’ perspectives.

### 2.5 Data collection and outcome measures

Data from the in-depth user sessions were captured using Zoom^®^ (2022 version, San Jose CA) video and audio recordings. Zoom^®^ video recordings allow for both the participant and the moderator’s monitor display to be recorded. The recordings were then transcribed using the transcribing software Grain^®^ (2021 version, San Francisco CA) and reviewed independently by two analysts (ND and NR) to extract suitable content for analysis. Three analysts (ND, BH, NR) and one pharmacogenomic specialist (EE) analyzed the first three sessions and codified the data to establish common themes, utilizing the qualitative data analysis software Nvivo^®^ (v11 plus, Denver CO). Our thematic analysis focused on specific sections of the consultation note (subjective, phenoconversion, assessment, plan, PGx table, flipped note concept, and general idea), reflecting the structure of the in-depth sessions. Table 1 provides definitions for each section. We evaluated each section based on strengths, weaknesses, and suggestions for improvement. After establishing common themes, multiple analysts independently reviewed each session. Disputes were resolved by a team of four analysts and a PGx specialist. To ensure rigor, at least three individuals reviewed each session. Finally, CSUQ data were presented quantitatively as mean, median and interquartile range.

**TABLE 1:** Main description of each section used for analysis.

Sections	Description
Subjective/Objective	Encompasses statements related to various sections, including the History of present illness, current medications affected by pharmacogenomic results, relevant pharmacogenetic test results, relevant labs, relevant drug interactions, and phenoconversion. Also includes statements about the outpatient EPIC-generated medication list and drug allergies
Phenoconversion	Involves statements regarding the overall clarity and ease of use of the “Clinical Phenotype” section of the note, along with its interpretation
Assessment	Encompasses statements regarding the “Test Results Interpretation” section within the Assessment section of the progress note
Plan	Involves statements made in regards to the “Plan” section of the note, including any feedback or observations related to this aspect
Flipped Note	Encompasses statements referring to a format of the progress note where the “Plan” section is positioned at the top of the note. Focuses on user feedback and perceptions of this format
PGx Table	Encompasses statements related to the PGx table found in the “Plan” section of the note. Feedback or comments specific to this table are included in this category
General	Encompasses statements that are nonspecific to a particular section. Includes overall recommendations or general feedback related to the entire note

## 3 Results

Table 2 displays the demographics of the study participants as well as their initial assessment with PGx knowlege. We sent out invitations to 79 potential participants between January 2022 and February 2022. Eleven providers were recruited and interviewed for the study (response rate 15%), but only 10 participants were included in the demographics analysis due to a data error (unretrievable) in one participant’s information. Data from this participant was still included by the rest of the analysis. Among the participants, three providers had a practice experience ranging from 10 to 14 years, while two providers had practiced for 20 years or more.

**TABLE 2:** Participant Demographics. Characteristics of the ten providers who participated in the in-depth user sessions.

Participant characteristics	Results *n* = 10, (%)
Female Sex (%)	3 (30)
Years of Practice (%)	
5–9	1 (10)
10–14	3 (30)
15–19	2 (20)
20 or more	2 (20)
*Self-Perception of PGx Knowledge* (*%*)	
Have some idea with PGx, however, does not know how to apply the information	2 (20)
Clear idea, however have not used PGx in practice	1 (10)
Can explain the concept of PGx, and is comfortable using it in their practice	7 (70)

^a^ Demographic data of one participant were corrupted and cannot be analyzed.

Regarding participants’ knowledge of PGx, the majority of providers (70%) expressed that they are comfortable applying their knowledge of PGx in their practice. Additionally, 20% stated that they had a conceptual understanding of the idea but faced challenges in applying the information.

**FIGURE 1:**
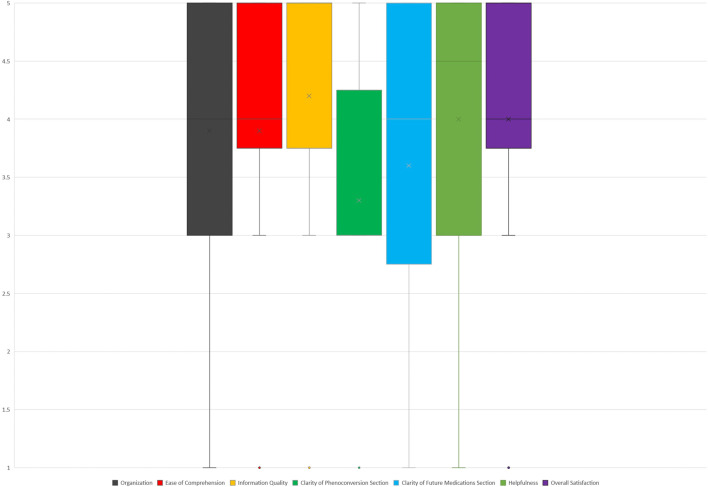
CSQU satisfaction data (1: strongly disagree, 5: strongly agree).

The CSUQ survey findings revealed that four out of ten providers strongly agreed with the statement indicating satisfaction with the note’s organization. On the other hand, six out of ten providers neither agreed nor disagreed regarding the clarity of the phenoconversion section, suggesting a potential opportunity for redesigning this specific section to enhance understanding and user experience (See Figure 1).

**FIGURE 2:**
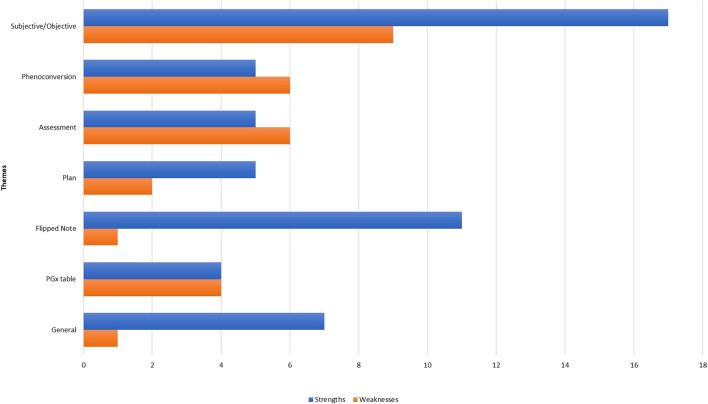
Quantifying participant comments based on type and appropriate section of the consult note.

Table 3 presents key themes and illustrative quotes extracted from the in-depth user interviews. It highlights strengths and weaknesses identified within each section of the consult note. Figure 2 quantifies participant feedback for each consult note section. Table 4 compiles significant suggestions from participants to improve the PGx consultation note’s design. Below is a summary of the main information collected for each section.

**TABLE 3:** Main themes and example quotes from participants.

Categories		Themes (number of participants)	Example quotes
Subjective/Objective	Strengths	General satisfaction with Subjective/Objective section. (Rosala, 2020)	“I think this is actually thorough, I do not think there’s anything that is missing from the subjective section.” —Participant 8 (session 5)
		EHR-generated medication list* is helpful. (Yen and Bakken, 2011)	“Yeah, I like that [EHR generated medication list] because otherwise you have to flip back to their chart. That is very pertinent in terms of interaction and influences how we’re going to prescribe— knowing what else they’re taking.“—Participant 5 (session 3)
		*Automatically generated by EHR when medications are ordered and/or completed by various healthcare providers completing medication reconcilliations	
		List of patient’s medications affected by the PGx results* (indication for the consult note) is useful. (Johnson et al., 2013)	“I think what’s listed is, is appropriate in the current medications affected by the results.“—Participant 7 (session 3)
		* Labeled as “Current medications affected by pharmacogenetic results:” on note	
		History of Present Illness (HPI) I section is satisfactory. (Johnson et al., 2013)	“I think this is fine.“—Participant 1 (session 1)
		Important to include allergies in Subjective/Objective section. (Johnson et al., 2013)	“Do you put allergies in here or no? [moderator answers yes] Okay yeah that’s an important one too.“—Participant 5 (session 3)
		Pharmacist generated medication list, using outside resources and interviewing patient directly, is helpful. (Yen and Bakken, 2011)	“I loved the one you have [pharmacist generated medication list] with why they stopped them and that kind of stuff. That I would actually use and read, but not the pre-populated one.“—Participant 3 (session 2)
	Weaknesses	Concern that pertinent labs (e.g., creatine, AST/ALT) may not be reliable or out of date, especially if provider looks back at note at a later date. (Johnson et al., 2013)	“I do fear that if the note is a little dated and there are more recent labs, people will References the listed labs rather than the current labs.“—Participant 9 (session 6)
		List of patient’s medications affected by the PGx results (indication for the consult note) is confusing. (Johnson et al., 2013)	“I found that to be confusing. I did not entirely understand if that is for newly prescribed medications that are sought for.“—Participant 11 (session 7)
		* Labeled as “Current medications affected by pharmacogenetic results:” on note	
		EHR generated list of patient medications is not viewed as reliable by providers. (Yen and Bakken, 2011)	“The other list, the pre-populated one, is not reliable at all. It depends on who was taking a med history and most of the time it’s completely wrong.“—Participant 2 (session 2)
		EHR generated medication list provides too much information and is not necessary for the note. (Johnson et al., 2013)	“If I saw a list like this it would not be super helpful.“—Participant 11 (session 7)
		Section that lists pertinent labs is unnecessary for note. (Johnson et al., 2013)	“That would be very non-meaningful. The only ones we pay attention to are creatinine since it plays a big part of post-surgical recovery.“—Participant 11 (session 7)
		Including the HPI makes the note too long and this information is available elsewhere in patient chart. (Johnson et al., 2013)	“It would just make the note longer because, you know, as a treating physician, that’s something that we would do anyways and probably know that from other sources.“—Participant 1 (session 1)
Phenoconversion	Strength	Content within phenoconversion section* is clear and useful. (Yen and Bakken, 2011)	“It’s clear, do not have anything I want to change about that section. It’s good as it is, simple for us that I would not change anything there.“—Participant 10 (session 7)
		* Labeled as “Relevant CYP___ Drug Interactions as of Date of note” within the note	
		Listing alternative medications to the medications affected by the PGx is helpful and relevant. (Johnson et al., 2013)	“What was helpful was listing the other alternatives that they can take.“—Participant 5 (session 3)
		Phenoconversion section is important to include. (Yen and Bakken, 2011)	“I think it’s the most important part because that’s our guide to prescribing.“—Participant 5 (session 3)
		Phenoconversion section is placed properly within the note. (Eadon et al., 2016)	“It seems to flow well where you have it.“—Participant 9 (session 6)
		Recommend keeping phenoconversion section near the top of the note. (Johnson et al., 2013)	“It should be pretty prominent near the top.“—Participant 11 (session 7)
		Concern over reliability of EMR to accurately convey a patient’s current medications. (Johnson et al., 2013)	“It’s helpful, the trick relies on your trust and belief in the current EMR. So, the trick is to get reliable input on the EMR active medications list, and you have a static document that lives in a dynamic world.“—Participant 11 (session 7)
	Weaknesses	Phenoconversion is not a well-understood concept at baseline for many providers. (Elchynski et al., 2021)	“Yeah, I would not say I’m as familiar either.“—Participant 4 (session 3)
		Phenoconversion section has poor visibility for providers. (Johnson et al., 2013)	“I think I would have it stand out more and draw my attention a little more to it.“—Participant 4 (session 3)
		Phenoconversion section is not important and should not be included. (Johnson et al., 2013)	“I do not like it. I do not think it’s helpful.“—Participant 2 (session 2)
		The phenoconversion section unclear for providers. (Johnson et al., 2013)	“I thought the sentence ‘adding/replacing drugs’ was a little unclear.“—Participant 8 (session 5)
		Phenotype (e.g., CYP3A4) not presented in an easily digestible way and is ignored by provider. (Eadon et al., 2016)	“I kind of gloss over the phenotype to be, to be honest with you and, and maybe I should not.“—Participant 4 (session3)
Assessment	Strength	Assessment section is satisfactory. (Elchynski et al., 2021)	“I love how this is done. It’s really clear about whether they’re controlled or uncontrolled and what considerations for the physician.“—Participant 7 (session 4)
	Weaknesses	Assessment section is not helpful. (Johnson et al., 2013)	“To me seems like a slightly less helpful section of the note than some of the others, or like that test results interpretation up above.“—Participant 11 (session 7)
		Assessment section should be more succinct. (Rosala, 2020)	I agree I think it’s a little bit heavy and redundant for this section.“—Participant 9 (session 6)
		Assessment should include all pertinent information that provider would use to make recommendation. (Johnson et al., 2013)	“For me, the assessment is a synthesis of everything that you’ve put together so far in this note. It’s lumping in the parts of the history that were important, the parts of the labs that were important, what helped you to make the recommendation that you’re going to make.” —Participant 3 (session 2)
		Listing the current regimen in assessment is not relevant. (Johnson et al., 2013)	“I thought listing the current regimen is not relevant in this section because it’s not part of the assessment.“—Participant 8 (session 5)
Plan	Strengths	The inclusion of alternatives in the plan section is appropriate. (Johnson et al., 2013)	“I did note that you said ‘switched to an alternative agent such as.’ I think that wording is appropriate. I think the clinician can always adjust accordingly.“—Participant 7 (session 4)
		Plan section is well received. (Yen and Bakken, 2011)	“And the plan was clear and concise.“—Participant 9 (session 6)
	Weaknesses	Listing allergies is not necessary. (Johnson et al., 2013)	“I think you can remove allergies, but nothing other than that.“—Participant 8 (session 5)
PGx Table	Strengths	Provider comfortable using the PGx table for future prescribing. (Johnson et al., 2013)	“I feel like when I look at this table, I’m approaching it from a perspective that this is a fixed patient response and not necessarily that it is modified by the note at the bottom.“—Participant 3 (session 2)
		PGx table is useful. (Rosala)	“I think it’s really interesting and helpful.“—Participant 9 (session 6)
	Weaknesses	PGx table contains too much information. (Rosala)	“My experience has shown that trying to scrutinize every single interaction can be cumbersome.“—Participant 2 (session 2)
		PGx table is not useful. (Johnson et al., 2013)	“So as a specialist, it’s not very helpful to me, not to say it’s bad, it’s of less clinical use to me.“—participant 11 (session 7)
Flipped Note	Strengths	Prefers flipped note format over standard. (Longo et al., 2021)	“Yeah, I’ve seen this [flipped note] use more and more. Especially when I’m attending a patient, it’s super helpful because often these patients are very complex and a lot of different consultants have adopted this model.“—participant 4 (session 3)
	Weaknesses	Prefers standard format over flipped. (Johnson et al., 2013)	“I think it’s really per person preference. I really do not care anymore. In this particular case, I think I would lean that way too [standard form], because it sort of tells a story and once someone’s seen this, once they know how it tells the story and they can spend however much time they feel they need to in each section.“—Participant 7 (session 4)
General	Strengths	Likes the specificity of the note template. (Johnson et al., 2013)	“We sometimes get some pharmacogenetics consult notes for studies were enrolled in and they’re not this specific, they’re much more general. And I wished that they were more specific like this.“— Participant 7 (session 4)
		Note template is consistent with other notes seen in practice. (Eadon et al., 2016)	“The note is structured well, so it’s pretty standardized.“—Participant 10 (session 7)
		Overall note template is good. (Eadon et al., 2016)	“I think the template overall it's good.“—Participant 5 (session 3)
		Structure of note template is good. (Eadon et al., 2016)	“I like how your notes are clear and separated into these little concise sections.“— Participant 5 (session 3)
	Weaknesses	Note is too long. (Johnson et al., 2013)	“Our notes are too saturated with non-relevant information. I would condense this. This is for PGx results and it’s 3 pages so it’s too much.“— Participant 10 (session 7)

**TABLE 4:** Major suggestions for improvement from participants.

Suggestion	Example quote
Include only relevant labs to medications affected by the PGx results	“I think it would have to be selective … it’s something [labs] that would influence what we would do with medication. Because sometimes you do not want the note to get too wordy. I like being able to go in look and see if you have enough information to know what their other meds are.“— Participant 5 (session 3)
Note should include list of present and past patient medications affected by PGx results	“I think it’s helpful because you can see the related conditions”—Participant 7 (session 4)
Tailor the HPI to the indication for the PGx consult note. (Eadon et al., 2016)	“I think having a more niche HPI would be nice like having a more of a medication history, just to show what the patient has used in the past.“—Participant 4 (session 3)
Include description of phenoconversion and recommendation. (Johnson et al., 2013)	“I think it’s helpful to have the description in there, but even more important for how it will impact medication use.“—Participant 7 (session 4)
Integrate note findings into EHR best practice alerts for abnormal results. (Johnson et al., 2013)	“Is there a way to integrate the phenoconversion data into the interaction checker in EPIC? Because I think that would be really neat if we could get this information within EPIC.“—Participant 3 (session 2)
Assessment should include alternative medications. (Johnson et al., 2013)	“This is where it would be helpful to have suggested alternatives that would be likely to have greater safety or effect profile with the phenotype that the patient is.“—Participant 11 (session 7)
Tailor PGx plan to physician specialty. (Eadon et al., 2016)	“I’d want the plan to be focused on that question [what was the consult for] but for us as a service that’s looking for guidance in one domain, it would be ultimately be the most useful to have the recommendation focused on that one domain.“—Participant 11 (session 7)
PGx table should have a phenoconversion column. (Johnson et al., 2013)	“I do think that table is helpful, and the additional column gives people an idea of how this applies today.“— Participant 7 (session 4)
Have a uniform note label so it can be easily searched for within EPIC. (Johnson et al., 2013)	“I think just having a uniform label you can search for across encounters.“—Participant 9 (session 6)
Recommended to add dispensed report (prescriptions from a variety of outpatient pharmacies)	“Is there a way you can tell which medication were dispensed? I heard you mentioning something like that, to look up what other pharmacies may be dispensed to the patient. It might be helpful to have that dispense report.“—Participant 2 (session 2)

### 3.1 Subjective/objective section

While participants expressed overall satisfaction with the section’s content, they provided valuable suggestions for enhancing the included information. Notably, in the History of Present Illness (HPI) section, Participant one suggested the importance of including the reason for the physician’s test request—an idea echoed by all sessions. Additionally, some providers expressed a preference for a more detailed HPI and an extensive medication history to showcase past patient use (Participant 4).

Our note incorporates both a pre-populated list from the Electronic Health Record (EHR) and a past medication list compiled by PGx pharmacists from patient information and external medication records. Participants unanimously found the pre-populated lists unreliable and strongly favored those generated by the pharmacist, particularly since the note encompasses both current and past medications.

Regarding the inclusion of laboratory information and genotype details, most providers disagreed, citing their availability in the EHR for review (Participant 8). Alternatively, some favored the inclusion of only pertinent labs to prevent the note from becoming overly verbose (Participant 5). Finally, there was a divergence of opinions on including allergies in the note. Two providers in session three emphasized the importance of listing patient allergies in this section, while two participants in session four argued that including allergies is unnecessary due to existing chart information and proposed its removal to streamline the note.

### 3.2 Phenoconversion

Phenoconversion, defined as the ability of external factors, such as drug-drug-interaction, to modify a predicted phenotypic expression base on genotype, is a crucial aspect (Shah and Smith, 2015; Klomp et al., 2020; Cicali et al., 2021). Although the clarity of content in this section was acknowledged, many participants were unfamiliar with the term “phenoconversion”, wf which was not explicitly mentioned in the note but surfaced during discussions. Several participants considered the information in this section paramount, emphasizing their tendency to immediately explore recommendations for potential medication changes based on the results (Participant 4). Ten out of the eleven participants found this information beneficial, with suggestions to enhance its visibility, such as making it stand out more or potentially segregating it into its own section (Participant 4).

While some participants felt that the phenoconversion information might be better placed in the assessment section, a few did not mind its repetition in several locations of the consult note, recognizing its importance to their clinical decision-making. One participant remarked on the insufficient information in this section, and Participant five proposed that listing alternative medications would be helpful for better guidance.

### 3.3 Flipped note

During each user sessions, two note formats were presented to participants, and their preferences were assessed. The flipped note format was favored by the majority, with ten out of eleven participants expressing a preference for it, while one participant remained neutral. The prevailing sentiment among participants was that the flipped note format is preferable due to its ability to prominently display essential information. Participant 11 highlighted its efficiency, stating that it is “really helpful because you’re cutting right to the chase,” especially in complex cases involving multiple consultants.

However, there was a dissenting opinion, as one participant preferred the standard format, citing its contribution to the logical flow of the patient’s story. Participant seven noted, “it is really a person preference. In this particular case, I think I would lean that way too [standard form], because it sort of tells a story.” The diverse preferences underscored the subjective nature of individual preferences in note formats, with some favoring efficiency and directness while others valued a narrative structure.

### 3.4 Assessment section

A significant critique of the assessment section was to improve the conciseness, with providers expressing that it was “a little bit heavy and redundant for this section” (participant 9) and that it “did not feel like it is an assessment and it felt like a history instead” (Participant 3). Participants favored familiar terms such as “well controlled or poorly controlled” and advocated for brevity, suggesting that the assessment should use as few terms as possible while still effectively summarizing the information from preceding sections. Additionally, participants recommended tailoring the assessment to the specific medication that prompted the consult and including only pertinent labs for a more streamlined and relevant summary.

### 3.5 Plan section

While all providers acknowledged the clarity and conciseness of this section, there were varying opinions on its necessity in the note. Some providers believed that this section was not essential, with three expressing the view that pharmacists should refrain from making clinical recommendations within the note. According to Participant 3, they were “looking for specific changes to medications” based on the pharmacogenomic (PGx) results. These providers voiced concerns about patient visibility of these notes, suggesting that PGx notes should primarily present relevant information for physicians to use in their broader clinical decision-making.

Another suggestion that emerged was the idea of tailoring the plan to the specialty of the provider who requested the note. This recommendation aimed at providing a more specialized and relevant plan, catering to the specific needs and context of the requesting healthcare professional.

### 3.6 PGx table

The note template concludes with a comprehensive list of other medications categorized by different indications that might be impacted by the patient’s CYP polymorphism. The intention behind this table is to offer providers a reference for future use, providing insights into potential medications affected by the patient’s polymorphisms. However, this table sparked the most discrepancies and differing opinions among the providers.

While seven providers found the table helpful and expressed comfort in using the information for future reference, four providers considered the table to be overly extensive and containing unnecessary details. Although most participants leaned towards a preference for a shorter table, others suggested additional information, such as including a list of alternative agents for each indication and incorporating a phenoconversion column. The diverse perspectives highlighted varying preferences regarding the level of detail and length of the table, emphasizing the need for customization to meet individual provider preferences and information needs.

### 3.7 General

Overall, participants expressed positive feedback regarding the PGx consultation note template’s consistency and alignment with other consultation note formats. This consistency fosters familiarity and ease of use within the broader EHR system. However, participants strongly recommended reducing the note’s length to improve efficiency and streamline information retrieval. Additionally, they emphasized the importance of using consistent titles for each note. This standardization would significantly enhance searchability within the EHR system, allowing clinicians to quickly locate specific PGx consultation notes and access relevant patient information.

## 4 Discussion

The implementation of pharmacogenomic (PGx) consultation services has become widespread across many institutions (Eadon et al., 2016; Bain et al., 2018; Longo et al., 2021). However, our study stands out as one of the first to adopt a user-centered approach for a formal assessment of provider needs and design requirements, aiming to guide future enhancements of PGx result notes. This approach allowed us to gain valuable insights into providers’ workflows and preferences regarding PGx information (Andreassen and Malling, 2019). Such knowledge is instrumental for PGx specialists in tailoring consult notes to align with the clinical context, facilitating easy navigation and utilization of relevant information. Several key concepts and ideas emerged from our study, providing valuable insights for institutions looking to implement or refine PGx services (see Table 4).

Responding to requests from specialists familiar with patients who sought a shortcut to the assessment section, we also offered PGx consult notes using the flipped format, where the assessment was presented first. Concerns about redundant information in consult notes have been reported in the literature (Brown et al., 2014; Huang et al., 2018), and our study received mixed comments on note format preferences. While several participants favored the standard SOAP note for its familiarity, others preferred a more concise version using the flipped note. Therefore, we recommend providing consultations using the SOAP format but offering flip notes as an option for providers with specific requests. The SOAP note format, being more traditional, is generally easier for new providers to comprehend. Similarly, for all other sections that receive mixed comments from participants, we compile a list and discuss them with our precision medicine leadership to discuss plan for implementation and prioritization.

Our note template also introduced a new section called phenoconversion, a crucial concept in PGx consult notes that might be overlooked or not fully understood by general healthcare providers. In our study, we identified that 60% of participants did not fully comprehend this concept, indicating a need for redesign for better clarification. We propose including a short description to define phenoconversion, aiding providers in better understanding this section. Additionally, it is essential to separate and clarify the differences between current active drug-gene interactions (DGI) and potential DGIs to prevent confusion. However, capturing the current active medication list for outpatients remains challenging due to current technological limitations which only able to capture medication order information but not medication dispensing records (Lin et al., 2021).

While consultation notes traditionally serve as a direct means of consultation for requested providers, leveraging technology can make information more accessible and extend recommendations to a broader pool of providers. The ability to provide succinct information emerged as a crucial theme in our study, prompting consideration for building a “genomics profile” within patients’ health record systems. The University of Florida Health has recently implemented this approach by incorporating the Epic^®^ Genomics Module. Utilizing technology from this module, we developed language capable of explaining and providing recommendations for each drug-gene interaction relevant to a specific patient profile. This genomics profile consolidates all relevant genetic information onto a single page, facilitating easy access for healthcare providers to make optimal prescribing decisions.

Despite these insights, our study has several limitations. We collected data from a single institution, and while UF Health is a large healthcare system, the workflow and structure may not be universally applicable to other institutions. Furthermore, the use of the Epic^®^ EHR at UF Health might not be representative of other EHR systems. Additionally, our study focused solely on physicians as the main requesters for PGx consult notes, and future research should consider collecting feedback from other healthcare providers, such as nurse practitioners or physician assistants. The use of in-depth user sessions, rather than one-on-one interview to collect feedback may lead to uneven contributions among participants, with some being more vocal than others. Lastly, our recruitment had a low response rate which might create a shwed representation of the target population.

### 4.1 Future directions

We plan to enhance the design of currently implemented PGx result note at our institution and disseminate a framework for other institutions who plan to implement PGx result documentation. Once we update the PGx note template, we plan to further evaluate the information provided in our PGx results note and provider satisfaction with the documentation through future provider interview. Ultimately, we will develop a practical design guideline to assist with PGx result documentation development as well as other consultation notes provided by pharmacists.

## 5 Conclusion

Utilizing provider feedback via in-depth user sessions and having providers complete the CSUQ regarding a PGx result note resulted in valuable feedback. The feedback collected will guide changes to the implemented PGx consult note at our institution and help create a standardized PGx consult note format.

## Data Availability

The raw data supporting the conclusion of this article will be made available by the authors, without undue reservation.
