# Supplementary material for: Assessing user perspectives on clinical pharmacogenomics consultation documentation: a user-centered evaluation
Source: Front Pharmacol. 2024 May 9;15:1377132. doi: 10.3389/fphar.2024.1377132 (PMC11111859; doi:10.3389/fphar.2024.1377132)
Supplement: Supplementary file 1 [file DataSheet2.PDF]

Supplemental documents

Supplemental document B:

Consult note templates used for each in-depth interview session

B1: Traditional PGx consult note template.

Pharmacogenetics Consultation
UF Health Precision Medicine Program

Subjective/Objective

HPI:
DK is a 82 y.o. male who underwent pharmacogenetic testing with the GatorPGx panel, based on patient's current use of the following medications;

Current medications affected by pharmacogenetic results:
Hydrocodone/acetaminophen 5-325 mg 1 tab q4-6 hr PRN
Pantoprazole 40 mg daily

Relevant* Pharmacogenetic Test Results:
- *CYP2C19 *1/*1* Normal metabolizer; normal CYP2C19 activity
- *CYP2D6 *2/*4* Normal metabolizer; normal CYP2D6 activity‡

B2: Flipped PGx consult note template.

Pharmacogenetics Consultation
UF Health Precision Medicine Program

Pharmacogenetic Test Results and Interpretation:
CYP2C19 (/*)* (Choose an item)

Recommendation:

HPI**:** [name] is a *** y.o. *** with a PMH significant for *** who ***

CYP2C19 testing was ordered (inpatient/outpatient) to guide current PPI therapy.

Future Medications Affected by CYP2C19:

Indication	Relevant Medications	CYP2C19 Choose an item. Phenotype Interpretation
Depression/Anxiety	Escitalopram	Choose an item.
