# Supplementary material for: Assessing user perspectives on clinical pharmacogenomics consultation documentation: a user-centered evaluation
Source: Front Pharmacol. 2024 May 9;15:1377132. doi: 10.3389/fphar.2024.1377132 (PMC11111859; doi:10.3389/fphar.2024.1377132)
Supplement: Supplementary file 2 [file DataSheet1.PDF]

Opening (introduction, example)

Hello,

Thank you for your participation in this focus group, we appreciate your valuable time and input.

My name is xxxxx clinical PGx pharmacy resident and I will be the moderator for today's session.

In this session, we have: Dr. xxxxx from cardiology department, Dr. xxxxxx from the orthopedic surgery department.

The purpose of this session is to get your feedback on the current format and content of the PGx consult notes. First, I will briefly provide background on the pharmacogenetic consult service and more directions will follow.

Upon return of PGx test results, PGx specialists open a 'Documentation Only' encounter within the patient's electronic health record and document their interpretation of the PGx results and any recommendations in a progress note. The encounter is then routed to the clinician who ordered and/or authorized the test.

UF Health Precision Medicine Program has periodically updated the templates used to create these notes. However, to date, there has never been a formal assessment of clinician needs to optimize the usability of the consult note.

In order to solicit your feedback on our consult note content and format, I will present a sample consult note and provide you with 5 min to review the note before moving to the questions. You will independently answer a brief survey assessing your satisfaction with the current consult note as a baseline. Once you complete the survey, you will convene to answer a series of questions I will ask verbally and assess your thoughts on it.

Different PGx consult note can be used based on specialty of each particpant

As mentioned there are several PGx consult notes that we used depending on the specialty ordering the test.

- Cardio and neurology note: CYP2C19-Clopidogrel
- GI note: CYP2C19-PPIs
- Psych note: CYP2D6/CYP2C19-SSRIs
- TPMT-Thiopurine
- PGx clinic note
- GatorPGx note: GatorPGx panel (7 genes)

Example of a PGx consultation note for DK

Pharmacogenetics Consultation

UF Health Precision Medicine Program

Subjective/Objective

HPI:

DK is a 82 y.o. male who underwent pharmacogenetic testing with the GatorPGx panel, based on patient's current use of the following medications;

Current medications affected by pharmacogenetic results:

Hydrocodone/acetaminophen 5-325 mg 1 tab q4-6 hr PRN

Pantoprazole 40 mg daily

Relevant* Pharmacogenetic Test Results:

- *CYP2C19 *1/*1* Normal metabolizer; normal CYP2C19 activity
- *CYP2D6 *2/*4* Normal metabolizer; normal CYP2D6 activity‡

**Additional pharmacogenetic test results are displayed in the Results Review tab under "Genetic Testing."*

Relevant CYP2D6 Drug Interactions as of 01/20/22

duloxetine

CYP2D6 Clinical Phenotype: Intermediate metabolizer

‡Because of a CYP2D6 drug interaction with duloxetine, this patient may resemble a Intermediate metabolizer and have decreased CYP2D6 activity while taking duloxetine. Replacing or adding one of the following drugs in the future may affect CYP2D6 clinical phenotype: bupropion, fluoxetine, duloxetine, paroxetine, abiraterone, cinacalcet, mirabegron, quinidine, terbinafine.

Allergies

Allergen	Reactions
• Lamotrigine	
• Miralax [Polyethylene Glycol]	
Pain andd cramps	
• Sulfa Antibiotics	Rash
• Trazodone	Other (See Comments)
Effects of medication did not subside. Pt loses his balance and falls long after effects were supposed to wear off	

Current Medications

Current Outpatient Medications

Medication	Sig	Dispense	Refill
• DULoxetine (CYMBALTA) 60 MG Oral Capsule Delayed Release Particles	Take 2 capsules by mouth daily for 90 days.	60 capsule	2
• meclizine (ANTIVERT) 12.5 MG Oral Tablet	Take 1 tablet by mouth 3 times daily as needed for dizziness or nausea.	30 tablet	1
• pantoprazole (PROTONIX) 40 MG Oral Tablet Delayed Release	TAKE 1 TABLET BY MOUTH EVERY MORNING 30 MINUTES BEFORE BREAKFAST	90 tablet	1
• amoxicillin-clavulanate (AUGMENTIN) 875-125 MG Oral Tablet	Take 1 tablet by mouth 2 times daily for 10 days.	20 tablet	0
• artificial tears Ophthalmic Ointment	as needed. Apply to eye(s) as needed		
• atorvastatin (LIPITOR) 10 MG Oral Tablet	TAKE 1 TABLET BY MOUTH DAILY	90 tablet	3
• Carboxymethylcellulose Sodium (Refresh Liquigel) 1 % Ophthalmic Gel	Apply 1-2 drops to eye(s) as needed for other (Blepharitis).		0
• cetirizine (ZyrTEC) 5 MG Oral Tablet	TAKE 1 TABLET BY MOUTH DAILY	30 tablet	11
• desonide (DESOWEN) 0.05 % External Cream	Apply topically 2 times daily	60 g	3
• Eyelid Cleansers (OCUSOFT LID SCRUB EX)	topically.		
• fluocinonide (LIDEX) 0.05 % External Solution	Apply topically 2 times daily	60 mL	3
• HYDROcodone-acetaminophen (NORCO) 5-325 MG Oral Tablet	Take 1 tablet by mouth every 6 hours as needed for pain. Reasons: Acute Pain (Patient not taking: Reported on 1/20/2022)	12 tablet	0

Another example of Subjective/objective since the previous example was short

Subjective/Objective:

HPI:

[redacted] is a 77 y.o. female who was referred to us by D[redacted] for anxiety, depression, multiple antidepressants failure. Patient presents to clinic to determine the need for pharmacogenetic testing related to her past use of citalopram, escitalopram, sertraline, and potential future use of sertraline, escitalopram, citalopram, paroxetine, and fluvoxamine.

Depression/anxiety

Patient is complaining from low mood and anxiety that are negatively impacting her daily life. She reported having some situational stressors in daily life including the upcoming shoulder replacement surgery and the recovery time after procedure. She reported that she tried multiple agents in the past without success. She attends therapy sessions, last one in Dec 2021, and reported it helps.

Current relevant medications

Buspirone 5 mg am and 10 mg pm (Aug 2020 - current). Anxiety not controlled and reported bothersome dizziness and headache.

Past medications

- **Escitalopram** 5 mg daily (March 2020 - April 2020): ineffective and couldn't recall side effects
- **Citalopram** 20 mg daily (Jan 2011 - Feb 2011): ineffective and couldn't recall side effects
- **Sertraline** 25 mg daily (Oct 2021 - Nov 2021): it caused her severe nausea for which she stopped using after 5 days
- **Bupropion** (back in 2010): she couldn't recall the regimen nor the efficacy/safety
- **Alprazolam** 0.5 mg TID PRN (Feb 2011 - March 2011): she couldn't recall efficacy/safety
- **Clonazepam** 0.5 mg BID PRN (July 2017 - Aug 2017): she couldn't recall efficacy/safety

Current medication

Indication	Relevant Medications	Clinical Phenotype Interpretation^Δ
Pain	Certain opioids<br>Codeine<br>Tramadol<br>Hydrocodone<br>Oxycodone	CYP2D6 IM: Little to no pain relief expected
	NSAIDS<br>Celecoxib<br>Flurbiprofen<br>Ibuprofen<br>Meloxicam<br>Piroxicam	CYP2C9 Normal Activity: Expected response
Nausea/ vomiting	Ondansetron	CYP2D6 IM: Very limited data available
ADHD	Atomoxetine	CYP2D6 IM: Increased chances of achieving therapeutic levels compared to normal metabolizers
Breast cancer	Tamoxifen	CYP2D6 IM: Increased risk of therapeutic failure, higher risk of breast cancer reoccurrence, event-free, and recurrence-free survival
Depression/ anxiety	SSRIs<br>Paroxetine<br>Fluvoxamine	CYP2D6 IM: Slightly increased risk of adverse effects
	Escitalopram<br>Citalopram<br>Sertraline	CYP2C19 NM: Normal response expected
GERD/H. Pylori/GI	PPIs<br>Dexlansoprazole<br>Esomeprazole<br>Lansoprazole<br>Omeprazole<br>Pantoprazole	CYP2C19 NM: Expected response
Fungal infection	Voriconazole	CYP2C19 NM: Expected response
Anticoagulation	Warfarin	Patient's genetic results, in combination with clinical factors, can predict initial warfarin dosing.
Transplant	Tacrolimus	CYP3A5 Non-Expresser: Increased chance of achieving target levels
Cardiology	Simvastatin	SLCO1B1 Normal Function: Normal response expected
	Clopidogrel	CYP2C19 NM: Expected response
Seizures	Phenytoin<br>Fosphenytoin	CYP2C9 Normal Activity: Normal phenytoin

ǂ<u>**NOTE**</u>**:** Because of a CYP2D6 drug interaction with duloxetine, this patient **may resemble a CYP2D6** intermediate metabolizer and have decreased **CYP2D6 activity while taking duloxetine.**

Review information from the assessment section

Assessment

Test results interpretation:

Based on CYP2D6 intermediate metabolizer phenotype it is predicted that the patient will have little to no pain relief with opioids (such as hydrocodone, codeine, and tramadol) and slightly increased risk of adverse effects with CYP2D6-mediated SSRIs (i.e., paroxetine, fluvoxamine)

Note:

If duloxetine stopped, patient will return to original CYP2D6 normal metabolizer phenotype which is associated with expected response with opioids (such as hydrocodone, codeine, and tramadol) and CYP2D6-mediated SSRIs (i.e., paroxetine, fluvoxamine)

Based on CYP2C19 normal metabolizer phenotype it is predicted that the patient will have normal response with CYP2C19-mediated SSRIs (i.e., sertraline, escitalopram, citalopram) and PPIs (such as omeprazole and pantoprazole).

Current depression regimen:

Duloxetine 120 mg daily

Patient saw psychiatry (12/10/21) and plan was to increase duloxetine to 120 mg daily and add quetiapine 50 mg at bedtime (to help with sleep). Patient is still on duloxetine 120 mg daily but quetiapine was interrupted because insurance didn't cover it. He reported his mood still haven changed as he rated his mood 4/10 (10 is the best). He reported that uncontrolled headache is a major contributing factor affecting his mood. He reported trying therapy and it helped and he will keep in mind continuing that.

Current pain regimen:

Hydrocodone/acetaminophen 5-325 mg 1 tab q4-6 hr PRN

Patient rates pain 9/10 at its worst, and on average 7/10). He failed multiple treatments in the past and no longer benefit with any interventional pain procedures. For which he was referred to neurosurgery and was put on 3 days course of hydrocodone until he sees them. He reported that hydrocodone helped alleviating his pain.

Current GERD regimen:

Pantoprazole 40 mg daily

Not controlled with pantoprazole 40 mg daily. He experienced relief with 40 mg BID however he is running out of supply as Rx is indicating 40 mg daily.

Review information for the plan section

Plan

Recommendation for current therapy:

- Switch duloxetine to an alternative antidepressant agent at an equivalent dose such as sertraline 100 mg daily and titrate up as clinically indicated. Please note, stopping duloxetine will be associated with CYP2D6 return to normal metabolizer phenotype (see interpretation above)
- Refer to pain management follow up after neurosurgery consultation to optimize ongoing headache pain management
- Increase pantoprazole to 40 mg BID as clinically indicated

Discussed the patient's GatorPGX panel(CYP2C19, CYP2D6, CYP2C9, CYP4F2, VKORC1, CYP3A5, SLCO1B1) results with him and the implications on hi antidepressant, opioid, PPI. We gave the patient a paper hand-out with an explanation of his GatorPGX panel(CYP2C19, CYP2D6, CYP2C9, CYP4F2, VKORC1, CYP3A5, SLCO1B1) results.

All questions answered; patient in agreement with the plan.

[redacted]

Time spent with patient: 60 minutes

Other medications:

Variability in the genes tested can impact the **effectiveness** and **toxicity** of certain medications.

For drug therapy recommendation for the relevant medications below, please re-consult the pharmacogenomics clinic. (REF853)

This is a different template for gastrointestinal specialty

GI consult note

Pharmacogenetics Consultation
UF Health Precision Medicine Program

Pharmacogenetic Test Results and Interpretation
*CYP2C19 (*1/*2)* (Intermediate metabolizer phenotype; decreased CYP2C19 activity) This genotype is associated with increased PPI levels and increased risk of adverse effects with chronic use.

Recommendation:
1. Switch **pantoprazole** 40 mg BID to an alternative PPI such as **Omeprazole** 20 mg BID 30- min before meal and monitor for GERD symptoms improvement.
   a. For chronic therapy (>12 weeks) and when efficacy achieved, consider 50% reduction in daily dose (**Omeprazole** 20 mg once daily) and monitor for continued efficacy
   b. Counsel patient on risk of adverse effects associated with chronic use of high dose PPI
2. If symptoms pressisted, consider adding an H2RA such as famotidine 20 mg at bedtime and assess for symptoms improvement.

HPI:
[redacted] 46 y.o. female with PMH significant for GERD and gastroparesis. During her recent visit (01/03/22) she mentioned that her symptoms are worsening (mainly heartburn and regurgitation) despite **pantoprazole** 40 mg daily. As for gastroparesis, she is positive for constipation and abdominal pain which are improving (no nausea or vomiting) and following gastroparesis diet loosely.

Current GERD regimen:
Pantoprazole 40 mg daily (2017- current) recently increased to 40 mg BID (01/03/22)

CYP2C19 testing was ordered outpatient to guide PPI therapy.

Future Medications Affected by CYP2C19:

Indication	Relevant Medications	CYP2C19 Intermediate metabolizer Phenotype Interpretation
Depression/Anxiety	Escitalopram<br>Citalopram<br>Sertraline	Slightly increased risk of adverse effects
Fungal Infection	Voriconazole	Normal response expected
Cardiology	Clopidogrel	Increased risk for major adverse cardiovascular events following PCI

We appreciate the opportunity to provide this consult. Please do not hesitate to ask if we can provide any further assistance:
EPIC message to: **P RX PMP MONITORING**
Phone number: **352-237-6415**

Hana Al Alshaykh, PharmD
Center for Pharmacogenomics and Precision Medicine
University of Florida | College of Pharmacy

Reference:
Lima JJ, Thomas CD, Barbarino J, et al. Clinical Pharmacogenetics Implementation Consortium (CPIC) Guideline for CYP2C19 and Proton Pump Inhibitor Dosing [published online ahead of print, 2020 Aug 8]. Clin Pharmacol Ther. 2020;10.1002/cpt.2015. doi:10.1002/cpt.2015

General questions (prompt questions)

- What parts would you like to add/remove to the note?
- How would you rearrange the note to have an ideal template?
- Do you have any ideas/suggestions to improve the note?

Use this example of PGx meds to ask question relate to this list

Indication	Relevant Medications	Clinical Phenotype Interpretation$^{\Delta}$	The original phenotype for CYP2D6 only
Pain	Certain opioids<br>Codeine<br>Tramadol<br>Hydrocodone<br>Oxycodone	CYP2D6 IM: Little to no pain relief expected	CYP2D6 NM: Normal response expected
	NSAIDS<br>Celecoxib<br>Flurbiprofen<br>Ibuprofen<br>Meloxicam<br>Piroxicam	CYP2C9 Normal Activity: Expected response	
Nausea/ vomiting	Ondansetron	CYP2D6 IM: Very limited data available	CYP2D6 NM: Normal response expected
ADHD	Atomoxetine	CYP2D6 IM: Increased chances of achieving therapeutic levels compared to normal metabolizers	CYP2D6 NM: Increased chance of treatment failure
Breast cancer	Tamoxifen	CYP2D6 IM: Increased risk of therapeutic failure, higher risk of breast cancer reoccurrence, event-free, and recurrence-free survival	CYP2D6 NM: Normal response expected
Depression/ anxiety	SSRIs<br>Paroxetine<br>Fluvoxamine	CYP2D6 IM: Slightly increased risk of adverse effects	CYP2D6 NM: Normal response expected
	Escitalopram<br>Citalopram<br>Sertraline	CYP2C19 NM: Normal response expected	
GERD/H. Pylori/GI	PPIs<br>Dexlansoprazole<br>Esomeprazole<br>Lansoprazole<br>Omeprazole<br>Pantoprazole	CYP2C19 NM: Expected response	
Fungal infection	Voriconazole	CYP2C19 NM: Expected response	
Anticoagulation	Warfarin	Patient's genetic results, in combination with clinical factors, can predict initial warfarin dosing.	
Transplant	Tacrolimus	CYP3A5 Non-Expresser: Increased chance of achieving target levels	
Cardiology	Simvastatin	SLCO1B1 Normal Function: Normal response expected	
	Clopidogrel	CYP2C19 NM: Expected response	
Seizures	Phenytoin<br>Fosphenytoin	CYP2C9 Normal Activity: Normal phenytoin metabolism	

Δ UM = ultrarapid metabolizer; RM = rapid metabolizer; NM = normal metabolizer; IM = intermediate metabolizer; PM = poor metabolizer
